# Integrated Immunohistochemical and Ultrastructural Characterization of Layer-Specific Capillary Specialization in the Human Vocal Fold

**DOI:** 10.3390/ijms27146193

**Published:** 2026-07-10

**Authors:** Roxana-Andreea Popa, Cosmin-Gabriel Popa, Delia Hînganu, Fabian Cezar Lupu, Cristinel Ionel Stan, Marius Valeriu Hînganu

**Affiliations:** 1 Department of Morpho-Functional Sciences I, Faculty of Medicine, Grigore T. Popa University of Medicine and Pharmacy, 700115 Iasi, Romania; roxana.popa@umfiasi.ro (R.-A.P.); cosmin-gabriel.popa@umfiasi.ro (C.-G.P.); cristinel.stan@umfiasi.ro (C.I.S.); marius.hinganu@umfiasi.ro (M.V.H.); 2 Department of Mechanical, Mechatronics and Robotics Engineering, Mechanical Engineering Faculty, “Gheorghe Asachi” Technical University of Iasi, 700050 Iasi, Romania; fabian-cezar.lupu@academic.tuiasi.ro

**Keywords:** true vocal fold, CD31, NSE, microvascular density, extracellular matrix, SEM, neurovascular microenvironment

## Abstract

The human true vocal fold exhibits a complex microvascular organization essential for its biomechanical and metabolic function. This study aimed to quantitatively assess CD31/PECAM-1-positive microvascular structures across the superficial lamina propria (SLP), deep lamina propria (DLP), and vocalis muscle (MV), and to integrate these findings with neuron-specific enolase (NSE) and scanning electron microscopy (SEM) observations. A retrospective analysis was performed on 21 formalin-fixed specimens. CD31 immunohistochemistry was used for endothelial identification, NSE immunohistochemistry was applied for the evaluation of neural elements, while SEM provided complementary ultrastructural information on extracellular matrix organization. Total microvascular density differed significantly among layers (χ^2^ = 32.12, df = 2, *p* = 1.06 × 10^−7^; Kendall’s W = 0.77), with highest values in MV (19.11 ± 6.22 vessels/field), followed by SLP (13.55 ± 3.93), and DLP (8.11 ± 2.41). Capillary density also showed significant inter-layer differences (*p* = 1.99 × 10^−7^), whereas small- and medium-caliber vessels did not (*p* = 0.081 and *p* = 0.538). NSE-positive neural profiles exhibited a similar distribution pattern, with higher density in the MV and lower values in the DLP. Inter-observer agreement was excellent (ICC = 0.91). Integrated analysis indicated a parallel spatial distribution of vascular, neural, and extracellular matrix components across vocal fold layers. This study provides a quantitative and structural baseline of the vocal fold microenvironment. This descriptive framework may inform future investigations of the layer-specific organization of vascular and neural-associated structures within the human vocal fold microenvironment.

## 1. Introduction

The true vocal folds are highly specialized anatomical structures whose biomechanical performance depends on the coordinated interaction between extracellular matrix composition, cellular components, neural regulation, and microvascular supply. Adequate vascularization is essential for maintaining tissue metabolism, oxygen delivery, and structural homeostasis in response to the repetitive mechanical stress generated during phonation [1,2,3]. Structurally, the vocal fold is organized into distinct anatomical layers with specific functional characteristics. According to Hirano’s classic body–cover model, the epithelium and superficial lamina propria constitute the pliable “cover,” whereas the deeper lamina propria and vocalis muscle form the more rigid “body” [4]. This layered organization underlies mucosal wave propagation and suggests the existence of region-specific vascular adaptations associated with different biomechanical and metabolic demands.

Previous histological and ultrastructural investigations have demonstrated the presence of a complex vascular network within the human vocal fold, particularly at the level of the lamina propria [5,6,7,8]. However, published data regarding the precise distribution and density of vascular structures across vocal fold layers remain heterogeneous. Some studies have reported increased vascularity within the superficial lamina propria, likely related to its role in vibratory function and susceptibility to mechanical stress [6], whereas others have described a more homogeneous vascular arrangement [7,9]. Such discrepancies may reflect differences in tissue processing, sampling strategies, and vessel identification criteria.

Immunohistochemical endothelial markers have significantly improved the characterization of microvascular organization beyond conventional hematoxylin–eosin evaluation. Among these markers, CD31 (platelet endothelial cell adhesion molecule-1, PECAM-1) is widely recognized as a reliable endothelial marker involved in endothelial integrity, leukocyte transmigration, angiogenesis, and vascular remodeling [10,11]. In addition to its structural role, PECAM-1 participates in mechanotransduction pathways that regulate endothelial responses to shear stress and mechanical loading [12,13,14,15,16,17]. Consequently, the spatial distribution of CD31-positive vascular structures may reflect regional endothelial adaptations associated with the functional specialization of the vocal fold layers.

In addition to vascular organization, vocal fold function is influenced by neural regulation and extracellular matrix architecture. Previous neurolaryngological and ultrastructural investigations have emphasized the importance of neural elements and stromal organization in maintaining tissue biomechanics and homeostasis [18,19]. Moreover, extracellular matrix composition and structural organization actively contribute to angiogenesis, tissue remodeling, and cell–matrix interactions [20]. Therefore, integrating vascular, neural, and extracellular matrix analyses may provide a more comprehensive understanding of the vocal fold microenvironment.

Despite previous histological, immunohistochemical, and ultrastructural investigations, quantitative layer-specific analyses integrating vascular, neural, and extracellular matrix organization remain limited. In particular, systematic morphometric studies distinguishing capillary density from larger vascular profiles across the superficial lamina propria, deep lamina propria, and vocalis muscle are scarce. Furthermore, the spatial relationship between vascular and neural components within the vocal fold microenvironment has not been sufficiently characterized.

The present study aimed to quantitatively characterize CD31-positive microvascular structures across the superficial lamina propria, deep lamina propria, and vocalis muscle using a standardized hotspot-based morphometric approach. In parallel, neuron-specific enolase (NSE) immunohistochemistry and scanning electron microscopy (SEM) were used to provide complementary information regarding neural and extracellular matrix organization. NSE was used as a broad neural-associated marker to provide complementary information regarding the regional distribution of neural elements. We hypothesized that the microvascular organization of the vocal fold is not homogeneous, but reflects the distinct biomechanical and metabolic requirements of its layered structure. The novelty of this study resides in the integrated quantitative evaluation of vascular, neural, and ultrastructural components within the human vocal fold microenvironment. By providing a structural baseline of morphologically histologically non-infiltrated tissue, this work may contribute to future investigations focused on angiogenesis, tissue repair, and pathological remodeling within the vocal fold.

## 2. Results

### 2.1. Histological Organization of the True Vocal Fold

**Figure 1:**
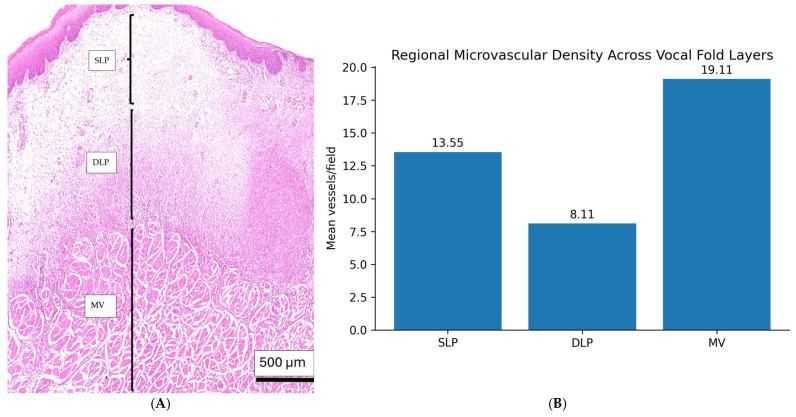
(**A**) Histological organization of the human true vocal fold. Representative hematoxylin–eosin (H&E) staining illustrating the layered structure of the true vocal fold. The epithelium overlies the superficial lamina propria (SLP), followed by the deep lamina propria (DLP) and the vocalis muscle (MV). Boundaries between regions were defined based on histological landmarks and guided region-specific quantitative analysis. Original magnification ×20. Scale bar: 500 μm. (**B**) Quantitative schematic representation of total regional vascular density across the superficial lamina propria (SLP), deep lamina propria (DLP), and vocalis muscle (MV). Values represent mean CD31-positive vessel density per microscopic field.

The layered organization of the true vocal fold was confirmed on hematoxylin–eosin-stained sections. The epithelium, SLP, DLP, and MV were clearly distinguishable based on established histological landmarks (Figure 1A). These anatomical regions served as the basis for subsequent region-specific quantitative analysis.

In addition to the histological overview, a schematic quantitative representation of regional vascular density across the vocal fold layers was incorporated to illustrate the anatomical framework used for subsequent morphometric analysis (Figure 1B).

### 2.2. CD31 Immunohistochemical Evaluation of Microvascular Distribution

**Figure 2:**
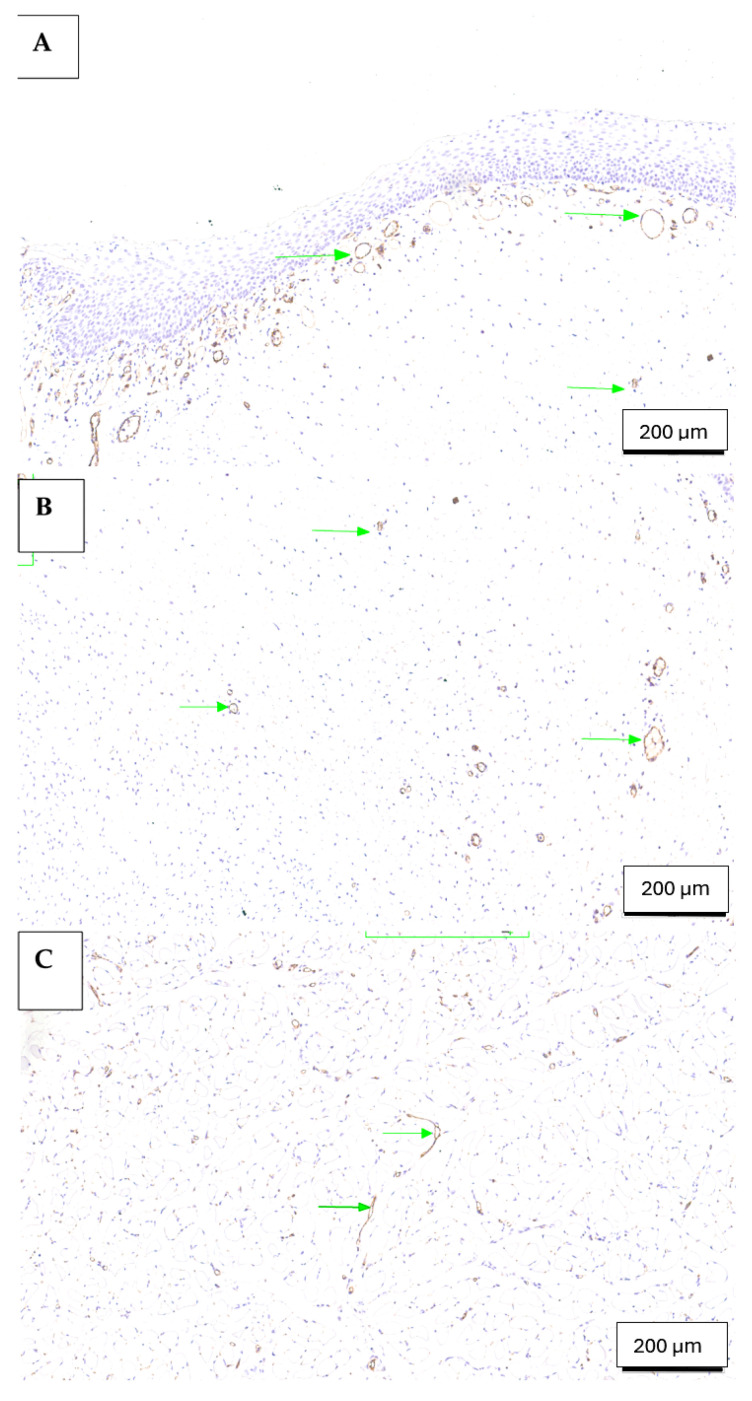

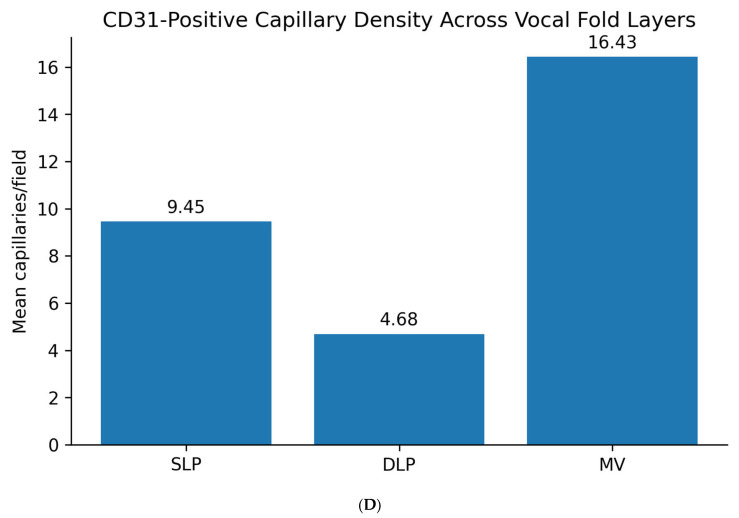
CD31 immunohistochemical staining in distinct vocal fold layers. Representative images showing CD31-positive vascular structures in the superficial lamina propria (SLP) (**A**), deep lamina propria (DLP) (**B**), and vocalis muscle (MV) (**C**). Endothelial-lined vessels are highlighted by membranous brown staining. Original magnification ×200. Scale bar: 200 μm. (**D**) Quantitative distribution of CD31-positive capillary density across vocal fold layers. The highest capillary density was observed in the vocalis muscle, followed by the superficial lamina propria and deep lamina propria.

CD31 immunohistochemistry demonstrated distinct membranous staining of endothelial cells outlining vascular lumina across all examined vocal fold layers (Figure 2). CD31-positive vascular structures were consistently identified in SLP, DLP, and MV, confirming the presence of an organized microvascular network throughout the tissue.

To complement the representative CD31 immunohistochemical images, quantitative graphical representation of hotspot-associated vascular density across the examined layers was included (Figure 2D), highlighting the regional differences subsequently confirmed by statistical analysis.

Qualitative assessment revealed apparent differences in hotspot-associated vascular density and distribution among the three regions, supporting subsequent quantitative analysis.

Inter-observer agreement was excellent, with an intraclass correlation coefficient (ICC) of 0.91, indicating high reproducibility of the morphometric measurements.

Additional representative CD31 immunohistochemical images from morphologically preserved control regions are provided in the Supplementary Materials (Figures S1–S3).

### 2.3. Distribution of Total Regional Microvascular Density Across Vocal Fold Layers

Quantitative analysis of CD31-positive vascular structures revealed significant differences in total hotspot-associated vascular density among the three vocal fold layers (Friedman test, χ^2^ = 32.12, df = 2, *p* = 1.06 × 10^−7^; Kendall’s W = 0.77).

The highest mean regional vascular density was observed in the vocalis muscle (MV) (19.11 ± 6.22 vessels/field), followed by the superficial lamina propria (SLP) (13.55 ± 3.93 vessels/field), while the deep lamina propria (DLP) exhibited the lowest values (8.11 ± 2.41 vessels/field) (Table 1).

The gradient of total regional vascular density followed the order MV > SLP > DLP.

Pairwise comparisons confirmed statistically significant differences between all regions, including SLP versus DLP (*p* = 1.20 × 10^−4^), SLP versus MV (*p* = 9.02 × 10^−4^), and DLP versus MV (*p* = 6.90 × 10^−5^).

### 2.4. Distribution of Vessel Size Categories Across Vocal Fold Layers

In addition to total regional vascular density, the distribution of vessel size categories was analyzed across the SLP, DLP, and MV.

**Figure 3:**
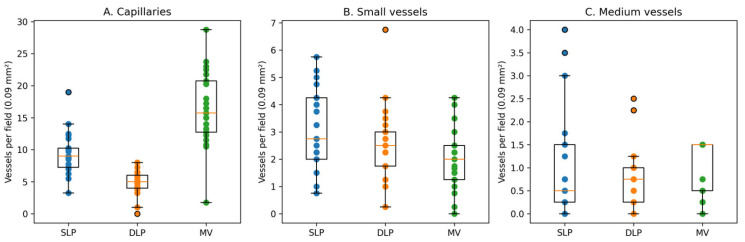
Distribution of capillary density (**A**), small-caliber vessels (**B**), and medium-caliber vessels (**C**) across vocal fold layers. Boxplots represent median, interquartile range, and range, with individual data points shown for each case. Capillary density differed significantly among layers (Friedman test, *p* = 1.99 × 10^−7^), whereas no significant differences were observed for small- or medium-caliber vessels.

Capillary density differed significantly among the three regions (Friedman test, *p* = 1.99 × 10^−7^), indicating a non-uniform distribution of capillaries across vocal fold layers. In contrast, no statistically significant differences were observed for small-caliber vessels (*p* = 0.081) or medium-caliber vessels (*p* = 0.538) (Figure 3; Table 2).

These findings indicate that layer-specific differences in microvascular organization are primarily driven by variations in capillary density, whereas the distribution of larger vascular profiles remains relatively uniform across anatomical regions.

In addition, neural elements were occasionally observed in close proximity to vascular structures, suggesting a potential spatial relationship between the two components.

### 2.5. NSE Immunohistochemical Evaluation

NSE immunohistochemistry demonstrated the presence of neural elements across all examined vocal fold layers, including the superficial lamina propria (SLP), deep lamina propria (DLP), and vocalis muscle (MV).

NSE-positive neural profiles were identified as discrete, well-defined structures exhibiting cytoplasmic brown staining, corresponding to nerve fibers and neural bundles of varying calibers. These elements were distributed throughout the extracellular matrix and, in some cases, were observed in close spatial proximity to CD31-positive vascular structures (Table 3).

**Figure 4:**
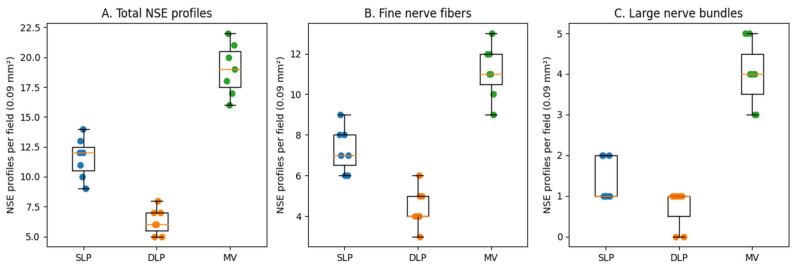
Distribution of NSE-positive neural profiles across vocal fold layers. (**A**) Total NSE-positive neural profiles per field. (**B**) Fine nerve fibers. (**C**) Large nerve bundles.

Quantitative assessment revealed a heterogeneous distribution of neural elements across the three anatomical regions. The highest density of NSE-positive neural profiles was observed in the MV, followed by the SLP, while the DLP exhibited the lowest neural density (Figure 4).

Boxplots represent the median, interquartile range, and range, with individual data points shown for each case. Neural profile density is expressed as the number of NSE-positive structures per microscopic field (0.09 mm^2^). The distribution pattern suggests a layer-specific organization of neural elements, paralleling the regional specialization observed for microvascular structures.

Morphological classification showed that fine nerve fibers predominated in the lamina propria, whereas larger and more organized nerve bundles were more frequently observed in the vocalis muscle.

**Figure 5:**
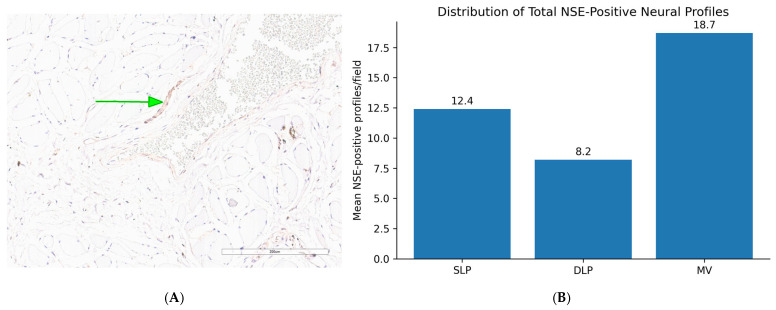
(**A**) Perivascular NSE-positive neural profile in the human vocal fold. NSE-positive neural elements are observed in close proximity to a vascular structure, suggesting a spatial relationship between neural and microvascular components. Neural profiles are visualized as brown cytoplasmic staining. Original magnification: ×200. Scale bar: 200 μm. (**B**) Quantitative distribution of total NSE-positive neural profiles across vocal fold layers. The highest neural density was observed in the vocalis muscle (MV), followed by the superficial lamina propria (SLP) and deep lamina propria (DLP).

**Figure 6:**
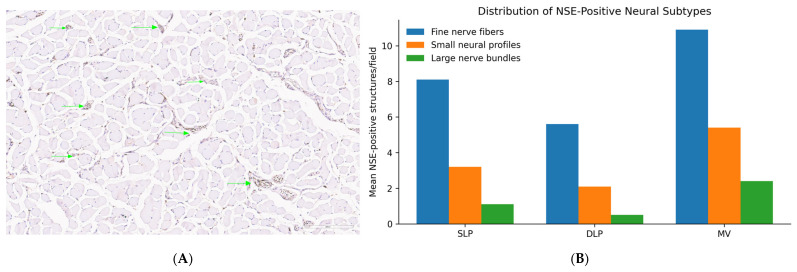
(**A**) NSE-positive neural elements of varying calibers in the vocal fold. Neural structures include fine nerve fibers and larger, more organized nerve bundles, demonstrating the heterogeneity of the neural component. NSE-positive elements are visualized as brown cytoplasmic staining. Original magnification: ×200. Scale bar: 200 μm. (**B**) Distribution of NSE-positive neural subtypes across vocal fold layers. Fine nerve fibers predominated in all regions, whereas larger neural bundles were more frequently observed in the vocalis muscle.

Representative NSE immunohistochemical images illustrating the distribution and morphological characteristics of neural elements in the vocal fold are shown in Figure 5 and Figure 6.

Quantitative graphical representation of NSE-positive neural profile distribution was added to complement the representative immunohistochemical images (Figure 5B and Figure 6B).

Additional representative NSE immunohistochemical images from morphologically preserved control regions are presented in the Supplementary Materials (Figures S4–S6).

### 2.6. Relationship Between Vascular and Neural Components

To explore the relationship between vascular and neural components, associations between CD31-defined regional vascular density and NSE-positive neural profile density were evaluated.

A positive association was observed between hotspot-associated vascular density and neural profile density across the examined regions, with higher values in the vocalis muscle and lower values in the deep lamina propria.

This distribution pattern suggests a parallel organization of vascular and neural elements within the vocal fold, consistent with the regional structural and functional specialization identified in the present study.

This observation was supported by a positive Spearman correlation between CD31-defined hotspot-associated vascular density and NSE-positive neural profile density.

### 2.7. Ultrastructural Organization of the Vocal Fold Microenvironment (SEM)

SEM provided complementary ultrastructural information regarding the organization of the extracellular matrix within the vocal fold.

SEM analysis revealed a complex, layer-dependent organization of the extracellular matrix, characterized by variations in fiber density, orientation, and spatial arrangement. At lower magnification, the extracellular matrix exhibited a heterogeneous architecture, while higher magnifications demonstrated a progressively more detailed fibrillar organization.

These ultrastructural features appear consistent with the regional differences observed in vascular and neural distribution, consistent the concept of a structurally integrated vocal fold microenvironment.

**Figure 7:**
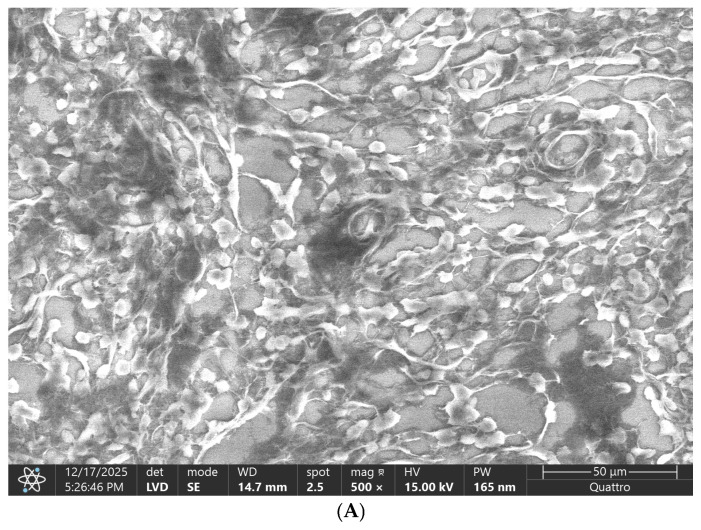

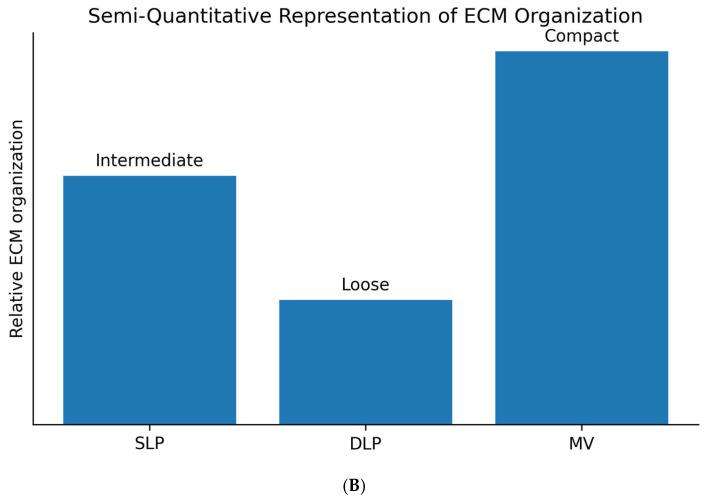
(**A**) SEM overview of extracellular matrix organization in the human vocal fold. (**B**) Semi-quantitative schematic representation of extracellular matrix (ECM) organization across vocal fold layers based on integrative SEM observations. The deep lamina propria (DLP) exhibited a comparatively looser fibrillar organization, whereas the vocalis muscle (MV) demonstrated a more compact stromal arrangement.

Low-magnification SEM image illustrating the general architecture of the extracellular matrix, showing a heterogeneous and regionally variable structural organization. Original magnification: ×100 (Figure 7).

**Figure 8:**
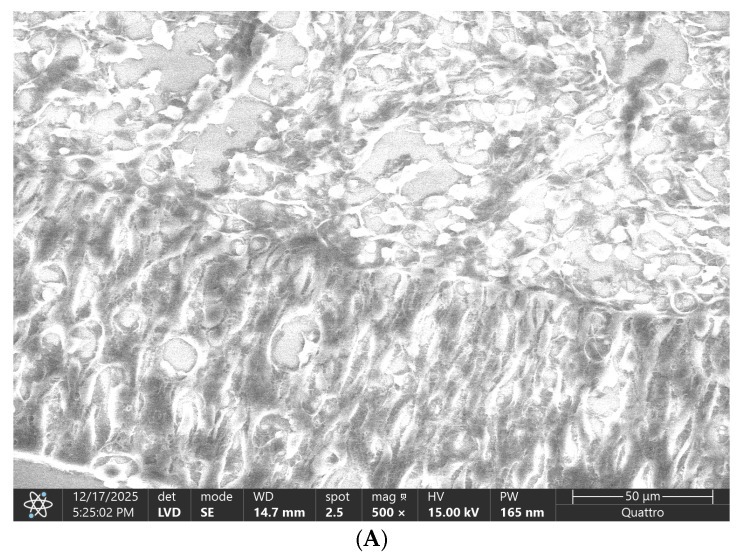

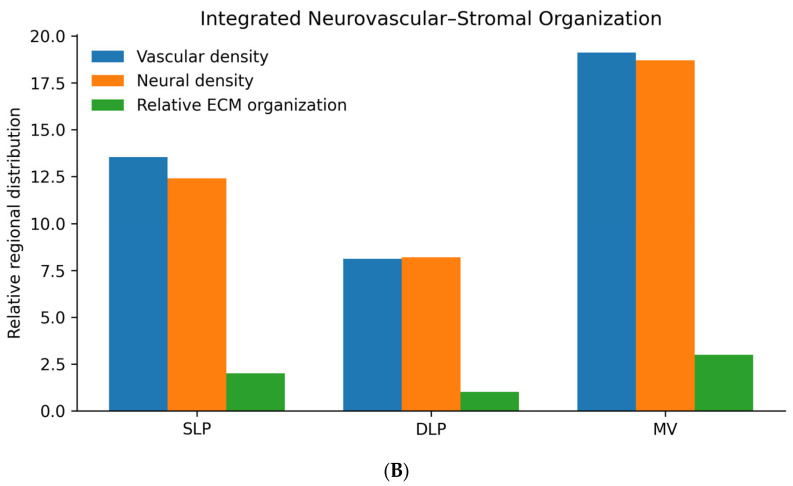
(**A**) Intermediate magnification SEM image of extracellular matrix organization. (**B**) Integrative schematic representation of the regional distribution of vascular, neural, and extracellular matrix components across vocal fold layers. The vocalis muscle exhibited the highest vascular and neural density associated with a more compact extracellular matrix organization.

The fibrillar network is more clearly defined, revealing the spatial arrangement and interconnection of extracellular matrix components. Original magnification: ×300 (Figure 8).

**Figure 9:**
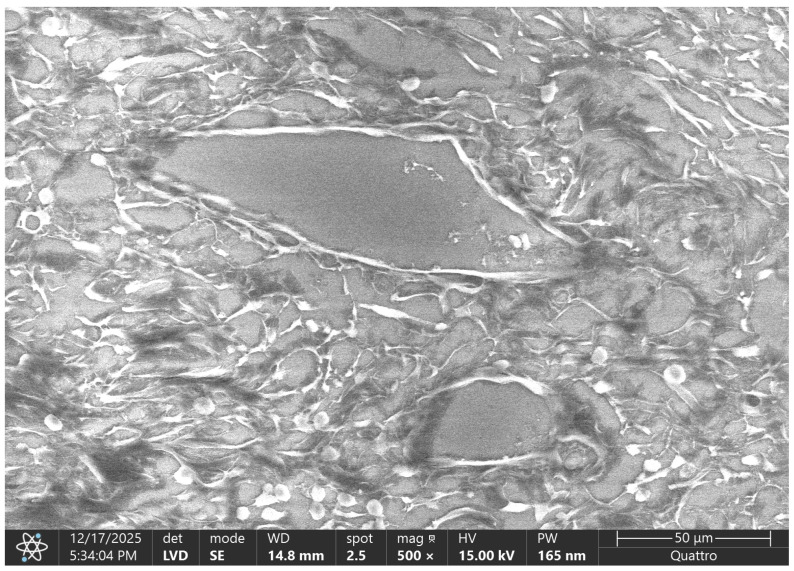
High-magnification SEM image showing ultrastructural details of the extracellular matrix.

Fine fibrillar structures and variations in matrix density are evident, highlighting the complexity of the ultrastructural organization. Original magnification: ×500 (Figure 9).

To improve integrative interpretation, semi-quantitative schematic representations summarizing regional extracellular matrix organization and its relationship with vascular and neural distribution were incorporated (Figure 7B and Figure 8B).

### 2.8. Integrated Analysis of Vascular, Neural, and Ultrastructural Findings

A combined evaluation of CD31 immunohistochemistry, NSE immunohistochemistry, and SEM observations was performed to assess the spatial relationship between vascular, neural, and extracellular matrix components of the vocal fold.

CD31-based quantitative analysis demonstrated a layer-specific gradient of regional vascular density, with highest values in the MV, followed by the SLP, and lowest values in the DLP. A similar distribution pattern was observed for NSE-positive neural profiles, with increased neural density in MV and reduced values in DLP.

Across all examined regions, neural elements were occasionally identified in close proximity to CD31-positive vascular structures on corresponding sections, indicating a spatial association between vascular and neural components.

SEM analysis revealed regionally variable extracellular matrix organization, characterized by differences in fibrillar density and structural arrangement. Areas exhibiting higher vascular and neural density were associated with a more compact and organized fibrillar architecture, whereas regions with lower CD31 and NSE values displayed a comparatively looser matrix organization.

These findings indicate a parallel spatial distribution of vascular, neural, and extracellular matrix components across vocal fold layers.

### 2.9. Layer-Specific Differences in Capillary Density: Post Hoc Analysis

Post hoc pairwise comparisons confirmed statistically significant differences in capillary density between all vocal fold layers, including SLP versus DLP (*p* = 9.22 × 10^−5^), SLP versus MV (*p* = 5.8 × 10^−4^), and DLP versus MV (*p* = 6.9 × 10^−5^).

These results demonstrate a consistent pattern of variation in capillary density across the examined anatomical regions.

### 2.10. Qualitative Assessment of the Non-Neoplastic Reference Cohort

Representative CD31 and NSE immunohistochemical images obtained from the non-neoplastic reference cohort are presented in Supplementary Figures S1–S6. These specimens originated from patients undergoing surgical treatment for bilateral vocal fold paralysis and were processed using the same histological and immunohistochemical protocols as the primary study cohort.

Qualitative examination confirmed the presence of preserved vascular and neural-associated structures with staining patterns comparable to those observed in morphologically preserved regions of the study cohort. Because of the limited amount of available tissue and the absence of a quantitatively matched sampling design, these specimens were used exclusively for structural reference and were not included in the statistical analyses.

## 3. Discussion

The hotspot-based methodology used in the present study was intentionally designed to evaluate regional maximal vascular and neural representation rather than average whole-section density. Consequently, the obtained values should be interpreted as indicators of local structural specialization and not as absolute tissue-wide quantitative estimates.

### 3.1. Principal Findings and Biological Significance

The present study provides a quantitative, layer-specific characterization of CD31/PECAM-1-positive microvascular structures in the human true vocal fold using a standardized morphometric approach. The principal findings can be summarized as follows: (i) total regional vascular density differs significantly among the SLP, DLP, and MV; (ii) these differences are primarily driven by variations in capillary density; and (iii) small- and medium-caliber vessels do not exhibit significant inter-layer variability.

These results demonstrate that the microvascular organization of the true vocal fold is not homogeneous but exhibits a clear layer-specific specialization. Importantly, the predominance of capillary-driven differences suggests that regional vascular adaptation may occur primarily at the level of the microcirculation, rather than through changes in larger vascular structures.

From a biological perspective, this pattern likely reflects the distinct biomechanical and metabolic demands of the vocal fold layers [2,21,22]. The superficial lamina propria, which plays a central role in mucosal wave propagation, is subjected to repetitive high-frequency mechanical stress and may require a finely tuned capillary network to sustain metabolic exchange and structural resilience. In contrast, the vocalis muscle, characterized by contractile activity, exhibits the highest overall hotspot-associated vascular density, consistent with the metabolic requirements of skeletal muscle tissue.

Beyond structural considerations, these findings have direct biomolecular implications. CD31/PECAM-1 is not only a marker of endothelial cells but also a key regulator of endothelial function, mediating cell–cell adhesion, leukocyte transmigration, and mechanotransduction [8,23]. The observed layer-specific differences in capillary density may reflect differential PECAM-1-associated endothelial responses to local mechanical forces, including shear stress and vibratory loading.

Taken together, the present data support a link between microvascular specialization of the vocal fold and both biomechanical function and endothelial signaling. Such organization may represent a structural substrate for differential responses to injury, angiogenesis, and extracellular matrix remodeling in vocal fold physiology and pathology.

The high inter-observer agreement observed in the present study further supports the robustness and reproducibility of the applied morphometric methodology.

### 3.2. Microvascular Specialization and Biomolecular Implications

The layer-specific differences in microvascular organization observed in the present study, particularly at the level of capillary density, suggest the presence of adaptive mechanism operating at the interface between mechanical forces and endothelial biology. In this context, mechanotransduction represents a key process through which endothelial cells sense and respond to biomechanical stimuli.

The true vocal fold is continuously exposed to repetitive vibratory stress during phonation, generating dynamic mechanical forces that may influence vascular behavior. Endothelial cells, through mechanosensitive molecules such as PECAM-1/CD31, are capable of detecting shear stress and translating it into intracellular signaling pathways that regulate vascular tone, angiogenesis, and structural remodeling [8,24,25].

The predominance of capillary-driven differences identified in this study suggests that microvascular adaptation in the vocal fold may primarily occur at the level of the microcirculation. Capillaries, due to their minimal diffusion distance and direct involvement in metabolic exchange, are particularly sensitive to local mechanical and metabolic conditions. Increased capillary density in specific layers may reflect enhanced metabolic demand, as well as an adaptive response to repetitive mechanical loading.

In addition to mechanotransduction, endothelial–stromal interactions may also contribute to the observed vascular patterns. Vocal fold fibroblasts and extracellular matrix components are known to modulate angiogenic signaling, influencing capillary formation and remodeling [26]. This interaction between the microvascular network and the surrounding tissue microenvironment, including perivascular support cells such as pericytes, may play a central role in maintaining vocal fold homeostasis [27].

From a biomolecular perspective, layer-specific vascular specialization may have important implications for tissue repair and pathological remodeling. Regions with higher capillary density may exhibit enhanced angiogenic potential and more efficient healing responses following injury, whereas areas with lower hotspot-associated vascular density may be more susceptible to hypoxia, delayed repair, or fibrotic changes. These processes are closely linked to endothelial signaling pathways and extracellular matrix dynamics, further supporting the relevance of microvascular organization in vocal fold biology.

### 3.3. Comparison with Previous Studies

Previous investigations have consistently described the presence of a well-developed microvascular network within the human vocal fold, particularly in the lamina propria [4,5,6,7,12,28]. However, most available data are derived from qualitative or semi-quantitative observations, and detailed layer-specific morphometric analyses remain limited.

The distribution of vascular structures across vocal fold layers has been variably reported. Some studies have suggested increased vascularity within the superficial lamina propria, likely reflecting its role in vibratory function and susceptibility to mechanical stress [5], whereas others have described a more uniform vascular arrangement [7,17]. Such discrepancies may be related to differences in methodology, including tissue processing, vessel identification criteria, and sampling strategies.

The present study provides quantitative evidence supporting a non-uniform, layer-specific microvascular organization. Importantly, by separately analyzing vessel size categories, this study shows that regional variation is predominantly driven by capillary density, whereas small- and medium-caliber vessels remain relatively constant across layers. These findings are consistent with the capillary-driven pattern identified in the present analysis. This pattern represents a key distinction from previous descriptive studies, which did not differentiate vascular subtypes in a systematic quantitative manner. This distinction has not been systematically addressed in most previous studies and may account for inconsistencies reported in the literature.

These findings are consistent with broader principles of microvascular biology, in which capillary networks represent the principal adaptive component of the vascular system, responding dynamically to local metabolic and mechanical demands [23,29]. In this context, the human vocal fold appears to follow general patterns of microvascular specialization, with capillary distribution serving as a key determinant of regional functional adaptation.

### 3.4. Integration of Vascular, Neural, and Extracellular Matrix Components in the Vocal Fold Microenvironment

In addition to the layer-specific microvascular organization demonstrated by CD31-based quantitative analysis, the present study complements these findings with neural (NSE) and ultrastructural (SEM) observations, allowing a descriptive characterization of the vocal fold microenvironment. The parallel distribution patterns observed for vascular and neural elements, with higher densities in the vocalis muscle and lower values in the deep lamina propria, suggest that these components are spatially coordinated rather than independently organized.

The neural-associated findings reported here should be interpreted in conjunction with our previous investigations of the intrinsic laryngeal neural network, in which neural connectivity and functional aspects were explored using dedicated experimental approaches. The present study was not designed to further characterize neural phenotypes but rather to provide complementary information regarding the spatial relationship between neural-associated structures, the microvascular compartment, and extracellular matrix organization within the vocal fold microenvironment [19].

Recent studies in laryngeal biology and neurolaryngology have emphasized the existence of complex neurovascular interactions within the vocal fold, including the presence of intrinsic neural networks capable of modulating muscular and vascular responses independently of central control mechanisms [30]. In this context, the proximity of NSE-positive neural elements to CD31-defined vascular structures observed in the present study represents a spatial association whose functional significance would require dedicated physiological or electrophysiological studies to elucidate.

Furthermore, SEM analysis revealed distinct differences in extracellular matrix organization across vocal fold layers, supporting the concept that the stromal component plays an active role in shaping tissue architecture. Emerging evidence indicates that extracellular matrix composition and organization are not merely structural but actively participate in regulating angiogenesis, neural guidance, and mechanotransduction through cell–matrix and cell–cell signaling pathways [24,31].

From a biomolecular perspective, the integration of vascular, neural, and extracellular matrix components aligns with current models of tissue microenvironments, in which endothelial cells, nerve fibers, and stromal elements form dynamic and interdependent systems [25]. Such interactions are particularly relevant in mechanically active tissues, where coordinated responses to stress are required to maintain structural integrity and functional performance.

Taken together, these findings support the concept that the vocal fold may be regarded as a structurally and functionally integrated microenvironment, in which vascular, neural, and extracellular matrix components exhibit coordinated spatial organization across anatomical layers.

Importantly, the present findings should be interpreted in direct continuity with our previously reported neurolaryngological observations, which suggested the existence of an intrinsic nervous network within the vocalis muscle. While the initial study primarily provided functional and neuroanatomical premises for this concept, the current investigation offers additional structural insight through the combined evaluation of NSE, CD31, and SEM features.

The close spatial association between neural elements and a well-defined microvascular network observed in this study is consistent with the morphological concept of a structured neurovascular microenvironment. Whether this arrangement contributes to metabolic support, modulatory interactions, or other functional roles relevant to intrinsic laryngeal function cannot be inferred from the present morphological data and would require dedicated functional investigations.

Although direct functional coupling cannot be demonstrated within the limits of the present study, these findings complement previous functional observations by providing additional structural information regarding the spatial organization of neural-associated and vascular elements by documenting the spatial organization of vascular components within the vocalis muscle, without implying functional regulatory mechanisms.

### 3.5. Clinical and Pathophysiological Implications

The identification of layer-specific differences in the microvascular organization of the true vocal fold, together with the parallel distribution of neural elements and ultrastructural matrix organization, may have relevant implications for both physiological adaptation and pathological processes. While capillary density represents a key determinant of regional vascular specialization, the present findings suggest that this parameter should be interpreted within a broader neurovascular–stromal context.

The superficial lamina propria, as the primary site of mucosal wave propagation, is particularly susceptible to repetitive vibratory trauma. In this context, not only an adequate capillary network, but also its spatial relationship with neural elements and extracellular matrix architecture, may be essential for maintaining tissue homeostasis and supporting efficient repair mechanisms. Alterations in any of these components may therefore influence healing dynamics and structural remodeling, including fibrosis or vocal fold scarring [16].

From a pathophysiological perspective, the coordinated organization of vascular, neural, and stromal components may contribute to the regulation of the local tissue microenvironment, including oxygen availability, angiogenic signaling, and potentially local modulatory interactions. These mechanisms may be relevant in the context of inflammatory processes and tissue remodeling and could contribute to the development of benign vocal fold lesions, such as polyps, although this relationship remains to be further investigated [12].

Importantly, as the present study was restricted to morphologically preserved tissue, the identified patterns should be interpreted as a baseline structural framework for future pathology-oriented studies. In this context, the observed spatial association between vascular and neural elements may provide structural observations relevant to future investigations of neurovascular organization within the vocal fold at the level of the vocalis muscle, potentially contributing to differential responses to injury and variability in pathological remodeling processes within the vocal fold.

### 3.6. Limitations

Although a non-neoplastic reference cohort was available for qualitative comparison, the primary quantitative analyses were performed on morphologically preserved vocal fold tissue obtained from laryngectomy specimens. The limited size and unilateral nature of the available non-neoplastic specimens did not allow the construction of a quantitatively matched control cohort. Therefore, the possibility of subclinical molecular alterations associated with field cancerization cannot be completely excluded.

The present findings demonstrate spatial relationships between vascular and neural-associated structures but do not establish functional communication or conduction mechanisms. Such questions will require dedicated physiological, electrophysiological, or tracer-based investigations.

First, the study cohort consisted of specimens obtained from total laryngectomies performed for unilateral neoplastic pathology. Although only morphologically preserved areas, distant from tumor involvement, were analyzed, subclinical microenvironmental alterations and potential field effects of the neoplastic process cannot be entirely excluded.

Another limitation of the study is related to the hotspot-based morphometric approach, which preferentially evaluates regions with maximal local vascular and neural density and therefore may not fully reflect the average distribution of these structures across the entire vocal fold tissue.

Second, the retrospective design limits control over demographic and clinical variables such as age, smoking status, or systemic vascular conditions, which may influence both vascular and neural tissue organization.

Third, the morphometric analysis was based on CD31 and NSE immunohistochemistry, allowing the identification and quantification of vascular and neural structures, but without providing functional information regarding blood flow, neural activity, or their potential interactions.

Additional antibody-specific validation approaches, including pre-absorption controls, were not performed; however, pre-absorption controls are not considered standard validation for commercially validated monoclonal antibody clones such as those used in the present study (CD31 clone JC/70A; NSE clone 22C9), where specificity has been established through extensive use in diagnostic pathology and research [32].

Furthermore, SEM analysis provided detailed ultrastructural information on extracellular matrix organization; however, it remains descriptive and does not allow direct assessment of dynamic or functional properties of the tissue microenvironment.

Another limitation of the present study is related to the use of NSE immunohistochemistry, as NSE is not entirely specific for neuronal tissue and may also be expressed in certain non-neuronal or neuroendocrine cell populations. Consequently, the neural findings should be interpreted as supportive morphologic observations rather than definitive neuronal characterization.

Finally, the present study is limited to structural and spatial observations. Therefore, any inference regarding coordinated neurovascular interactions or the existence of an integrated conduction model should be considered hypothetical and requires further validation through functional and experimental studies.

Although rigorous histopathological selection and oncological safety criteria were applied, the possibility of subclinical molecular alterations associated with field cancerization cannot be entirely excluded.

## 4. Materials and Methods

### 4.1. Study Design and Case Selection

True vocal fold specimens were obtained retrospectively from formalin-fixed, paraffin-embedded tissue blocks archived in the Department of Pathology of Sfântul Spiridon Clinical Emergency Hospital, Iași, Romania. The material originated from total laryngectomies performed for unilateral laryngeal neoplastic pathology. An initial cohort of 248 cases diagnosed between 2017 and 2025 was reviewed, from which 29 total laryngectomy cases were identified. Histological slides were re-evaluated, and 21 cases were selected based on the presence of well-preserved true vocal fold tissue without histological evidence of tumor involvement. Only morphologically preserved vocal fold areas located at a distance from the neoplastic lesion were included in the analysis. Tissue sampling was restricted to regions beyond accepted oncological resection margins (R0), ensuring the absence of tumor infiltration and minimizing the potential influence of tumor-associated microenvironmental changes.

All analyzed tissue fragments were selected from regions located beyond accepted oncological safety margins (R0 resection areas) and were independently re-evaluated histopathologically to exclude tumor infiltration, epithelial dysplasia, reactive stromal alterations, or inflammatory changes [21].

The demographic characteristics of the study cohort are summarized in Table 4.

Inclusion criteria comprised the availability of intact true vocal fold tissue free of tumor infiltration and adequate preservation for histological and immunohistochemical evaluation. Exclusion criteria included bilateral tumor involvement, direct tumor extension to the vocal fold, extensive inflammatory alterations, tissue autolysis, or insufficient material for comprehensive analysis. The study was conducted in accordance with the Declaration of Helsinki and was approved by the Ethics Committee of Grigore T. Popa University of Medicine and Pharmacy of Iași (protocol code 304; approval date: 16 May 2023). Representative immunohistochemical images from morphologically preserved control regions used for structural validation are provided in the Supplementary Materials (Figures S1–S6).

#### Reference Non-Neoplastic Cohort

In addition to the primary study cohort, a non-neoplastic reference cohort consisting of eight patients was available for qualitative comparative assessment. These specimens were obtained from patients undergoing surgery for bilateral vocal fold paralysis, including posterior cordectomy (Kashima procedure) and/or arytenoidectomy performed to enlarge the airway. None of these patients had a history of laryngeal neoplasia. Excised tissue fragments were processed using the same fixation, histological, immunohistochemical, and imaging protocols applied to the main study cohort.

Because these procedures provide only limited unilateral tissue samples and are relatively uncommon in our institution, the available material was used as a qualitative reference group for structural validation and representative supplementary documentation rather than as a quantitatively matched control cohort.

### 4.2. Tissue Processing and Histological Evaluation

Tissue samples were fixed in 10% neutral buffered formalin for 24–48 h, routinely processed through graded ethanol dehydration, cleared in xylene, and embedded in paraffin. Serial sections of 4–5 μm thickness were obtained using a rotary microtome and mounted on glass slides. For general morphological assessment and identification of anatomical layers, sections were stained with hematoxylin and eosin (H&E) according to standard laboratory protocols. H&E-stained sections were examined to evaluate epithelial integrity and to define the boundaries of the superficial lamina propria (SLP), deep lamina propria (DLP), and vocalis muscle (MV).

All slides were digitally scanned using a Leica Aperio whole-slide imaging system (Leica Biosystems, Wetzlar, Germany) at ×20 magnification. Digital evaluation was performed using ImageScope software, version 12.4.6 (Leica Biosystems, Wetzlar, Germany), ensuring standardized visualization and consistent morphometric assessment across all cases. To improve reproducibility, vessel classification and counting were independently performed by two observers, and discrepancies were resolved by joint review and consensus. Inter-observer reproducibility was evaluated by independent assessment of randomly selected cases by two blinded observers, and agreement was quantified using the intraclass correlation coefficient (ICC).

All measurements were performed on digitally scanned slides using the same magnification settings and predefined morphologic classification criteria. Borderline structures were reviewed independently by two observers and resolved by consensus.

### 4.3. CD31 Immunohistochemistry

Endothelial cells were identified using CD31/PECAM-1 immunohistochemistry. Briefly, 4–5 μm-thick formalin-fixed, paraffin-embedded tissue sections were deparaffinized in xylene, rehydrated through graded ethanol solutions, and rinsed in distilled water. Antigen retrieval was performed using citrate buffer, pH 6.0, for 20 min, followed by cooling at room temperature. Endogenous peroxidase activity was blocked according to the detection kit protocol. Sections were incubated overnight at 4–6 °C with a mouse monoclonal anti-CD31 antibody (clone JC/70A, Abcam, Cambridge, UK) diluted 1:500.

Immunodetection was performed using a polymer-based horseradish peroxidase/DAB detection system (Mouse and Rabbit Specific HRP/DAB Detection Kit, ab64264, Abcam, Cambridge, UK), according to the manufacturer’s instructions. Visualization was achieved using 3,3′-diaminobenzidine (DAB) as chromogen, followed by hematoxylin counterstaining. Sections were dehydrated, cleared, and mounted for microscopic evaluation. Vessel-rich tissue sections with known CD31 expression were used as positive controls, while negative controls were obtained by omission of the primary antibody.

### 4.4. NSE Immunohistochemistry

To evaluate the neural component of the vocal fold microenvironment, immunohistochemical staining for neuron-specific enolase (NSE) was performed on representative formalin-fixed, paraffin-embedded sections. Sections of 4–5 μm thickness were deparaffinized in xylene, rehydrated through graded ethanol solutions, and rinsed in distilled water. Antigen retrieval was performed using retrieval buffer at pH 9, according to the manufacturer’s recommendations for the antibody system used. Endogenous peroxidase activity was blocked according to the detection protocol.

Sections were incubated with a ready-to-use mouse monoclonal anti-NSE antibody (clone 22C9, Leica Biosystems) according to the manufacturer’s protocol. Immunodetection was performed using a polymer-based horseradish peroxidase/DAB detection system, with 3,3′-diaminobenzidine as chromogen, followed by hematoxylin counterstaining. Sections were dehydrated, cleared, and mounted for microscopic evaluation. Positive controls consisted of tissue sections with known NSE expression, whereas negative controls were obtained by omission of the primary antibody. NSE-positive structures were identified as neural elements, including nerve fibers and neural profiles within the lamina propria and vocalis muscle.

Because NSE is considered a broad neural-associated marker rather than a fully neuron-specific marker, its interpretation was limited to descriptive morphologic evaluation of neural-associated structures within the vocal fold microenvironment.

### 4.5. Quantitative Assessment of Vascular and Neural Density

CD31-positive vascular structures were quantitatively assessed using a standardized hotspot-based morphometric approach. Hotspot selection was performed according to established principles of vascular morphometry by identifying areas with the highest density of CD31-positive vascular structures, allowing the evaluation of maximal regional microvascular representation rather than average vascularization. For each specimen, the true vocal fold was subdivided into three anatomical regions: superficial lamina propria (SLP), deep lamina propria (DLP), and vocalis muscle (MV), based on histological landmarks identified on H&E-stained sections. Within each region, four non-overlapping square fields measuring 300 × 300 μm (0.09 mm^2^) were selected at ×20 magnification from hotspot areas, resulting in a total analyzed area of 0.36 mm^2^ per region per specimen. All CD31-positive vascular profiles with a clearly discernible endothelial lining were counted, and only vessels with their geometric center located within field boundaries were included to minimize edge bias.

The quantitative parameters included in the statistical analysis were: (i) total CD31-positive vascular density, (ii) capillary density, (iii) small-caliber vessel density, (iv) medium-caliber vessel density, (v) total NSE-positive neural profile density, and (vi) distribution of neural profile subtypes across the examined anatomical regions.

Vascular structures were morphologically classified into three categories using semiquantitative morphologic criteria based on estimated vessel caliber, luminal appearance, and vascular wall organization: capillaries, defined as CD31-positive vessels composed of a single endothelial layer without a visible muscular component (typically <10 μm in diameter); small-caliber vessels, characterized by a wider lumen and thin vascular wall without identifiable smooth muscle layers (approximately 10–30 μm); and medium-caliber vessels, exhibiting a larger lumen and a discernible muscular wall composed of one to two smooth muscle layers (approximately 30–100 μm). For each field, the total number of CD31-positive vessels and the number of vessels in each category were recorded, and regional mean values were calculated by averaging counts from the four analyzed fields. In parallel, NSE-positive neural elements were assessed using the same regional subdivision and hotspot-based methodology. Neural profiles were identified as discrete NSE-positive structures with cytoplasmic brown staining morphologically consistent with neural-associated elements and were semiquantitatively classified as fine nerve fibers, small neural profiles, or large nerve bundles based on relative structure size, organizational pattern, and staining appearance observed at standardized magnification settings. Mean values were calculated in the same manner as for vascular analysis.

All measurements were performed using the same magnification, field dimensions, hotspot selection criteria, and morphologic classification rules across all analyzed specimens. Hotspot selection was performed according to established principles of vascular morphometry by identifying areas with the highest density of CD31-positive vascular structures, allowing the evaluation of maximal regional microvascular representation rather than average whole-tissue vascular density. The same hotspot-based strategy was applied for NSE-positive neural elements in order to evaluate regional maximal neural representation pattern.

### 4.6. Scanning Electron Microscopy (SEM)

Scanning electron microscopy (SEM) was used to provide complementary ultrastructural information regarding extracellular matrix organization within the vocal fold microenvironment. Tissue samples obtained from the same cohort included in the histological and immunohistochemical analyses were fixed in 2.5% glutaraldehyde buffered with 0.1 M sodium cacodylate (pH 7.4), post-fixed in 1% osmium tetroxide, and dehydrated through graded ethanol solutions. Critical-point drying using carbon dioxide (CO_2_) was subsequently performed, followed by mounting on aluminum stubs and sputter coating with a thin gold layer (approximately 5–10 nm) to ensure electrical conductivity. Ultrastructural examination was carried out using a VegaTescan LMH II scanning electron microscope (Tescan, Czech Republic) operating under high-vacuum conditions at an accelerating voltage of 5–15 kV. Secondary electron detection was used for surface morphology evaluation. In addition, selected H&E-stained sections were adapted for SEM analysis through a deparaffinization and reprocessing protocol, enabling three-dimensional visualization of tissue architecture. SEM findings were used exclusively for qualitative and integrative interpretation and were not included in the quantitative statistical analysis.

### 4.7. Statistical Analysis

Statistical analysis was performed using SPSS software (version 31.0.0). Data distribution was evaluated using descriptive statistics, including skewness and kurtosis. Because at least one variable deviated from normal distribution, non-parametric statistical methods were applied. Differences in CD31-defined hotspot-associated vascular density among the superficial lamina propria, deep lamina propria, and vocalis muscle were assessed using the Friedman test for repeated measures, and effect size was calculated using Kendall’s W coefficient. When significant differences were identified, pairwise comparisons were performed using the Wilcoxon signed-rank test with Bonferroni correction. Associations between vascular and neural parameters were evaluated using Spearman’s rank correlation coefficient where applicable. SEM-derived observations were not included in the statistical analysis and were used exclusively for qualitative interpretation. A *p*-value < 0.05 was considered statistically significant.

## 5. Conclusions

The present study demonstrates that the microvascular organization of the human true vocal fold exhibits clear layer-specific variation, with significant differences in hotspot-associated vascular density among the superficial lamina propria, deep lamina propria, and vocalis muscle. These differences are primarily driven by capillary distribution, whereas larger vascular profiles remain comparatively uniform across layers. In parallel, NSE-positive neural-associated elements and SEM-derived extracellular matrix organization exhibited similar regional distribution patterns, supporting the concept of a spatially organized neurovascular–stromal microenvironment within the vocal fold. From a biomolecular perspective, this organization may reflect localized endothelial adaptation to mechanical stress associated with PECAM-1/CD31-related signaling mechanisms. By providing an integrated quantitative and structural characterization of morphologically preserved vocal fold tissue, this study establishes a descriptive reference framework for future investigations focused on angiogenesis, tissue repair, and pathological remodeling. Although the observed spatial associations may suggest biologically coordinated regional organization, the present findings remain fundamentally morphologic in nature, and any functional implications require further experimental validation.

## Figures and Tables

**Table 1:** Descriptive statistics of total regional vascular density assessed by CD31 immunohistochemistry in the superficial lamina propria (SLP), deep lamina propria (DLP), and vocalis muscle (MV).

Vocal Fold Layer	Mean ± SD (Vessels/Field)	Min–Max (Vessels/Field)
SLP	13.55 ± 3.93	7.25–25
DLP	8.11 ± 2.41	3.75–13
MV	19.11 ± 6.22	6.25–31

Data are expressed as the mean number of CD31-positive vessels per microscopic field (300 × 300 μm). For each specimen, values represent the average of four non-overlapping hotspot fields per region.

**Table 2:** Distribution of CD31-positive vascular structures by vessel size category across vocal fold layers.

Vocal Fold Layer	Capillaries (Mean ± SD)	Small-Caliber Vessels (Mean ± SD)	Medium-Caliber Vessels (Mean ± SD)
SLP	9.45 ± 3.33	3.08 ± 1.50	1.13 ± 1.34
DLP	4.68 ± 2.03	2.55 ± 1.40	0.87 ± 0.71
MV	16.43 ± 5.95	2.08 ± 1.21	0.98 ± 0.59

Data are expressed as mean ± standard deviation (SD) of vessel counts per microscopic field (0.09 mm^2^), calculated as the average of four non-overlapping hotspot fields per region per specimen.

**Table 3:** Distribution of NSE-positive neural profiles.

Parameter	SLP (Mean ± SD)	DLP (Mean ± SD)	MV (Mean ± SD)	*p*-Value
Total NSE profiles	12.4 ± 3.1	8.2 ± 2.5	18.7 ± 4.0	0.002
Fine nerve fibers	8.1 ± 2.2	5.6 ± 1.8	10.9 ± 2.7	0.005
Small neural profiles	3.2 ± 1.1	2.1 ± 0.9	5.4 ± 1.6	0.010
Large nerve bundles	1.1 ± 0.5	0.5 ± 0.3	2.4 ± 0.8	0.003

Data are expressed as mean ± standard deviation. Statistical analysis was performed using the Friedman test for repeated measures, with post hoc pairwise comparisons where applicable. A *p*-value < 0.05 was considered statistically significant.

**Table 4:** Demographic characteristics of the study cohort.

Case	Sample ID	Sex	Age	Environment	Year
1	194396	M	51	Rural	2017
2	202089	M	59	Urban	2017
3	206843	M	59	Rural	2017
4	212340	M	62	Rural	2018
5	268583	M	57	Urban	2022
6	268796	M	66	Urban	2022
7	269493	M	62	Rural	2022
8	270705	M	67	Urban	2022
9	272021	F	66	Rural	2022
10	277180	M	67	Urban	2022
11	291432	M	57	Rural	2023
12	297557	M	56	Rural	2023
13	308598	M	65	Urban	2024
14	313463	M	59	Rural	2024
15	200631	M	72	Urban	2017
16	211969	M	55	Rural	2018
17	227909	F	75	Rural	2018
18	240166	M	58	Rural	2019
19	256640	M	63	Urban	2021
20	263582	M	56	Rural	2021
21	300709	M	55	Urban	2024

Abbreviations: M, male; F, female.

## Data Availability

Data are contained within the article.

## Supplementary Materials

The following supporting information can be downloaded at: https://www.mdpi.com/article/10.3390/ijms27146193/s1.

## Abbreviations

The following abbreviations are used in this manuscript:

SLP	Superficial Lamina Propria
DLP	Deep Lamina Propria
MV	Vocalis Muscle
CD31	Cluster of Differentiation 31
PECAM-1	Platelet Endothelial Cell Adhesion Molecule-1
NSE	Neuron-Specific Enolase
H&E	Hematoxylin and Eosin
DAB	3,3′-Diaminobenzidine
SEM	Scanning Electron Microscopy
IHC	Immunohistochemistry

## References

[1] Gray S.D., Titze I.R., Chan R.W., Hammond T.H. (1999). Vocal fold proteoglycans and their influence on biomechanics. Laryngoscope.

[2] Michiels C. (2003). Endothelial cell functions. J. Cell Physiol..

[3] Hînganu M.V., Hînganu D., Cozma S.R., Asimionoaiei-Simionescu C., Scutariu I.A., Ionesi D.S., Haba D. (2018). Morphofunctional evaluation of buccopharyngeal space using three-dimensional cone-beam computed tomography (3D-CBCT). Ann. Anat..

[4] Hirano M. (1974). Morphological structure of the vocal cord as a vibrator and its variations. Folia Phoniatr. Logop..

[5] Newman S.R., Butler J., Hammond E.H., Gray S.D. (2000). Preliminary report on the histology of the human vocal fold. J. Voice.

[6] Sato K., Hirano M. (1995). Histologic investigation of the macula flava of the human vocal fold. Ann. Otol. Rhinol. Laryngol..

[7] Voigt-Zimmermann S., Arens C. (2015). Vascular lesions of vocal folds—Part 1: Horizontal vascular lesions. HNO.

[8] Kutta H., Steven P., Tillmann B., Paulsen F. (2004). The human vocal fold microvasculature: An ultrastructural study. Laryngoscope.

[9] Tracicaru R.V., Bräuer L., Döllinger M., Schicht M., Tillmann B., Hînganu D., Hristian L., Hînganu M.V., Paulsen F. (2024). Morphological evidence for a unique neuromuscular functional unit of the human vocalis muscle. Int. J. Mol. Sci..

[10] DeLisser H.M., Newman P.J., Albelda S.M. (1994). Molecular and functional aspects of PECAM-1/CD31. Immunol. Today.

[11] Lertkiatmongkol P., Liao D., Mei H., Hu Y., Newman P.J. (2016). Endothelial functions of platelet/endothelial cell adhesion molecule-1 (CD31). Curr. Opin. Hematol..

[12] Tzima E., Irani-Tehrani M., Kiosses W.B., Dejana E., Schultz D.A., Engelhardt B., Cao G., DeLisser H., Schwartz M.A. (2005). A mechanosensory complex that mediates the endothelial cell response to fluid shear stress. Nature.

[13] Popa R.-A., Popa C.-G., Hînganu D., Hînganu M.V. (2025). Microvascularization of the vocal folds: Molecular architecture, functional insights, and personalized research perspectives. J. Pers. Med..

[14] Tateda Y., Ikeda R., Kakuta R., Ono J., Izuhara K., Ogawa T., Ise K., Shimada H., Murakami K., Nakamura Y. (2022). Expression of periostin in vocal fold polyps. Tohoku J. Exp. Med..

[15] Fu T., Sullivan D.P., Gonzalez A.M., Haynes M.E., Dalal P.J., Rutledge N.S., Tierney A.L., Yescas J.A., Weber E.W., Muller W.A. (2023). Mechanotransduction via endothelial adhesion molecule CD31 initiates transmigration and reveals a role for VEGFR2 in diapedesis. Immunity.

[16] Gráczer É., Pászty K., Harsányi L., Lehoczky C., Fülöp A., Varga A. (2024). BRAF modulates the interplay between cell–cell and cell–extracellular matrix adhesions in PECAM-1-mediated mechanotransduction. Int. J. Mol. Sci..

[17] Bless D.M., Welham N.V. (2010). Characterization of vocal fold scar formation. J. Voice.

[18] Hînganu M.V., Cozma R.S., Ciochina P., Scutariu I.A., Asimionoaiei-Simionescu C., Hînganu D. (2017). The morphometry of the laryngeal phonatory system—Base of the anatomical study of the voice aptitudes. Rom. J. Morphol. Embryol..

[19] Tracicaru R.-V., Brauer L., Dollinger M., Tamba B.I., Szilagyi A., Catalin C.C., Hinganu D., Paulsen F., Hinganu M.V. (2024). Coactivation of the laryngeal muscles in pigs without external neural control indicates existence of an intrinsic neuronal network. J. Voice.

[20] Li L., Stiadle J.M., Lau H.K., Zerdoum A.B., Jia X., Thibeault S.L., Kiick K.L. (2016). Tissue engineering-based therapeutic strategies for vocal fold repair and regeneration. Biomaterials.

[21] Bernard S.E., van Lanschot C.G.F., Hardillo J.A., Monserez D.A., Meeuwis C.A., Baatenburg de Jong R.J., Koljenović S., Sewnaik A. (2024). A New Proposal for Adequate Resection Margins in Larynx and Hypopharynx Tumor Surgery—Are the RCP Guidelines Feasible?. Cancers.

[22] Cochereau T., Bailly L., Orgéas L., Henrich Bernardoni N., Robert Y., Terrien M. (2020). Mechanics of human vocal folds layers during finite strains in tension, compression and shear. J. Biomech..

[23] Levendoski E.E., Sivasankar M., Munoz-del-Rio A., Leydon C. (2014). Vocal fold epithelial barrier in health and injury: A review of cellular and molecular mechanisms. J. Speech Lang. Hear. Res..

[24] Dudley A.C., Griffioen A.W. (2023). Pathological angiogenesis: Mechanisms and therapeutic strategies. Angiogenesis.

[25] Power G., Ferreira-Santos L., Martinez-Lemus L.A., Padilla J. (2024). Integrating molecular and cellular components of endothelial shear stress mechanotransduction. Am. J. Physiol. Heart Circ. Physiol..

[26] Chu Y., Fang Y., Wu H., Chen J., Cheng L. (2022). Vocal fold fibroblasts promote angiogenesis in vocal fold leukoplakia by secreting pro-angiogenic factors. Auris Nasus Larynx.

[27] Sato K. (2018). Pericytes in the human vocal fold mucosa. Adv. Exp. Med. Biol..

[28] Sato K., Chitose S.-I., Sato F., Sato K., Ono T., Umeno H. (2023). Vascularity in the macula flava of human vocal fold as a stem cell niche. Auris Nasus Larynx.

[29] Chen Q., Jiang L., Li C., Hu D., Bu J., Cai D., Du J. (2023). The role of blood flow in vessel remodeling and its regulatory mechanism during developmental angiogenesis. Cell. Mol. Life Sci..

[30] Sweeney M., Foldes G. (2018). It takes two: Endothelial–perivascular cell cross-talk in vascular development and disease. Front. Cardiovasc. Med..

[31] Theocharis A.D., Skandalis S.S., Gialeli C., Karamanos N.K. (2016). Extracellular matrix structure. Adv. Drug Deliv. Rev..

[32] Burry R.W. (2011). Controls for immunocytochemistry: An update. J. Histochem. Cytochem..

