# Supplementary material for: Integrated Immunohistochemical and Ultrastructural Characterization of Layer-Specific Capillary Specialization in the Human Vocal Fold
Source: Int J Mol Sci. 2026 Jul 10;27(14):6193. doi: 10.3390/ijms27146193 (PMC13411090; doi:10.3390/ijms27146193)
Supplement: Supplementary file 1 [file ijms-27-06193-s001.zip › Supplementary figures S1-S6.pdf]

Supplementary Materials: Figures S1–S6 showing representative CD31 and NSE immunohistochemical images from morphologically preserved vocal fold tissue selected beyond oncological safety margins.

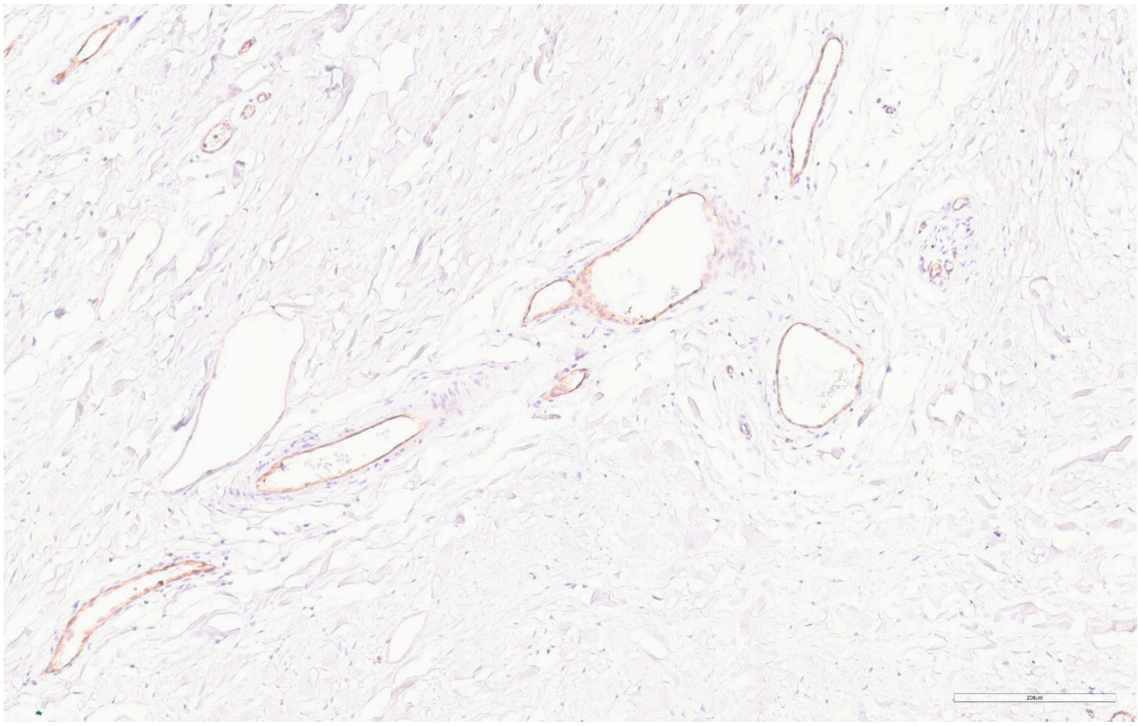

Figure S1. Representative CD31 immunohistochemical appearance of morphologically preserved vocal fold tissue. The image illustrates CD31-positive endothelial-lined vascular structures within the deep lamina propria (DLP) of the human true vocal fold. Brown membranous staining highlights vascular endothelial cells, confirming the presence of an organized microvascular network in histologically non-infiltrated tissue. The analyzed region was selected beyond accepted oncological safety margins and was histopathologically re-evaluated to exclude tumor infiltration or reactive alterations. Original magnification: ×200.

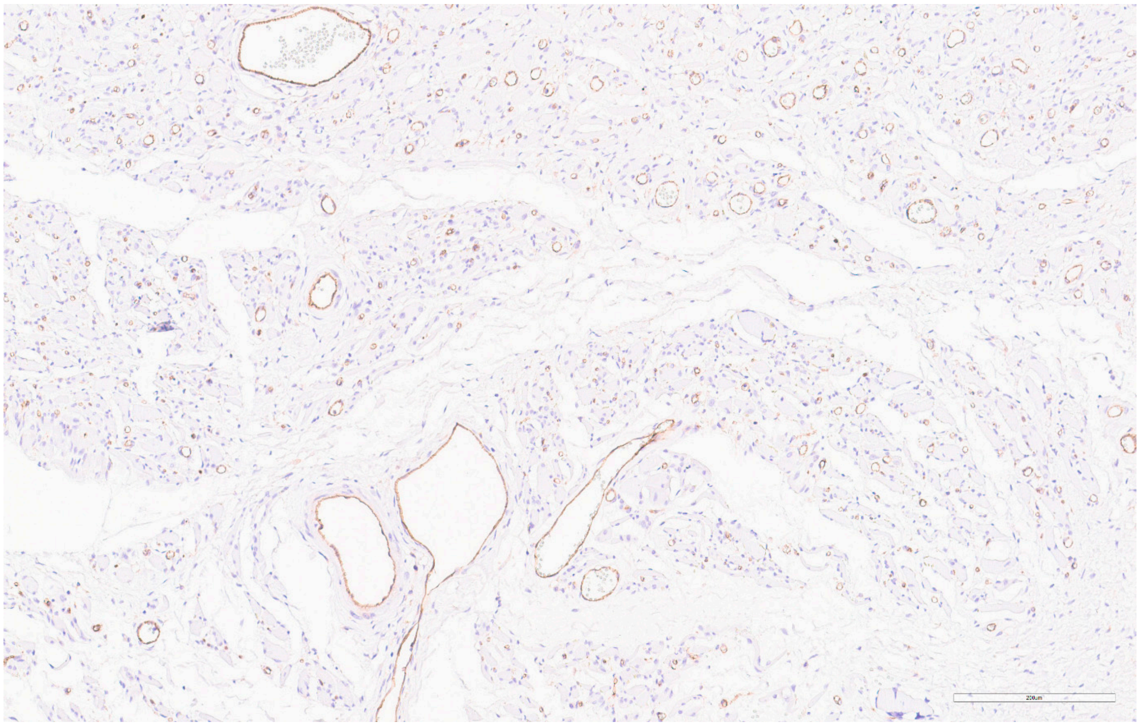

Figure S2. Representative CD31 immunohistochemical appearance of morphologically preserved vocal fold tissue. The image illustrates CD31-positive endothelial-lined vascular structures within the vocalis muscle (MV) of the human true vocal fold. Brown membranous staining highlights vascular endothelial cells and demonstrates the increased vascular density characteristic of the muscular compartment. The analyzed region was selected beyond accepted oncological safety margins and was histopathologically re-evaluated to exclude tumor infiltration or reactive alterations. Original magnification: ×200.

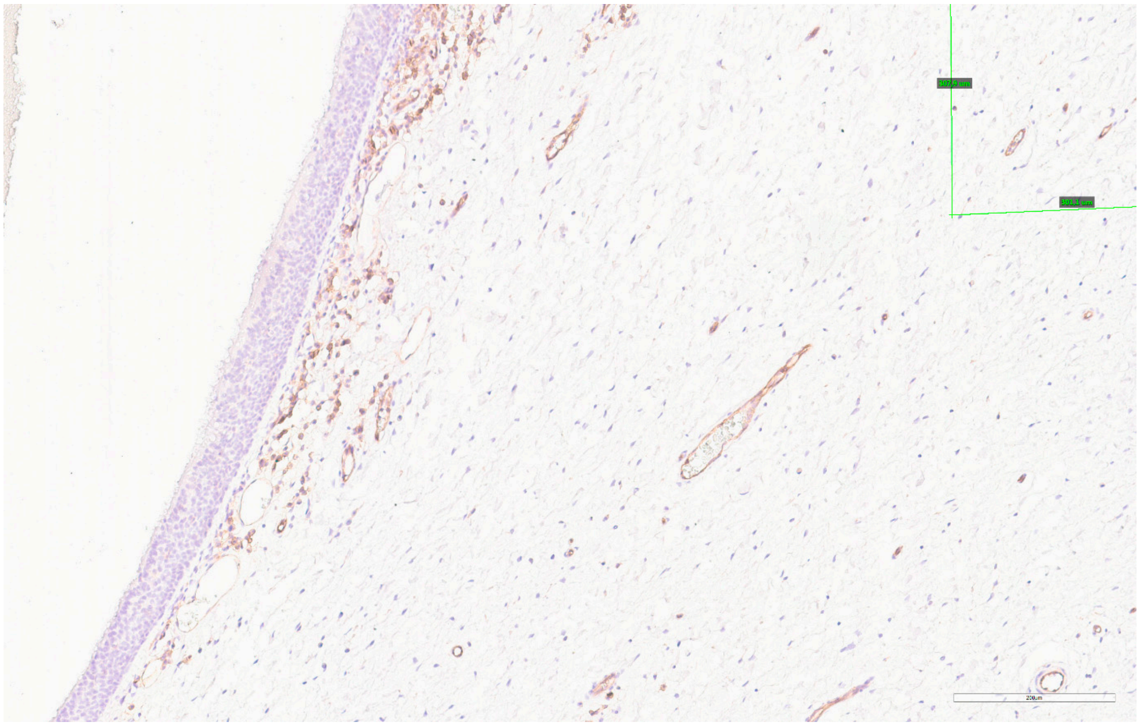

Figure S3. Representative CD31 immunohistochemical appearance of morphologically preserved vocal fold tissue. The image demonstrates CD31-positive endothelial-lined vascular structures within the superficial lamina propria (SLP) of the human true vocal fold. Brown membranous staining delineates endothelial cells outlining small vascular profiles distributed within the superficial stromal compartment. The analyzed region was selected beyond accepted oncological safety margins and was histopathologically re-evaluated to exclude tumor infiltration or reactive alterations. Original magnification: ×200.

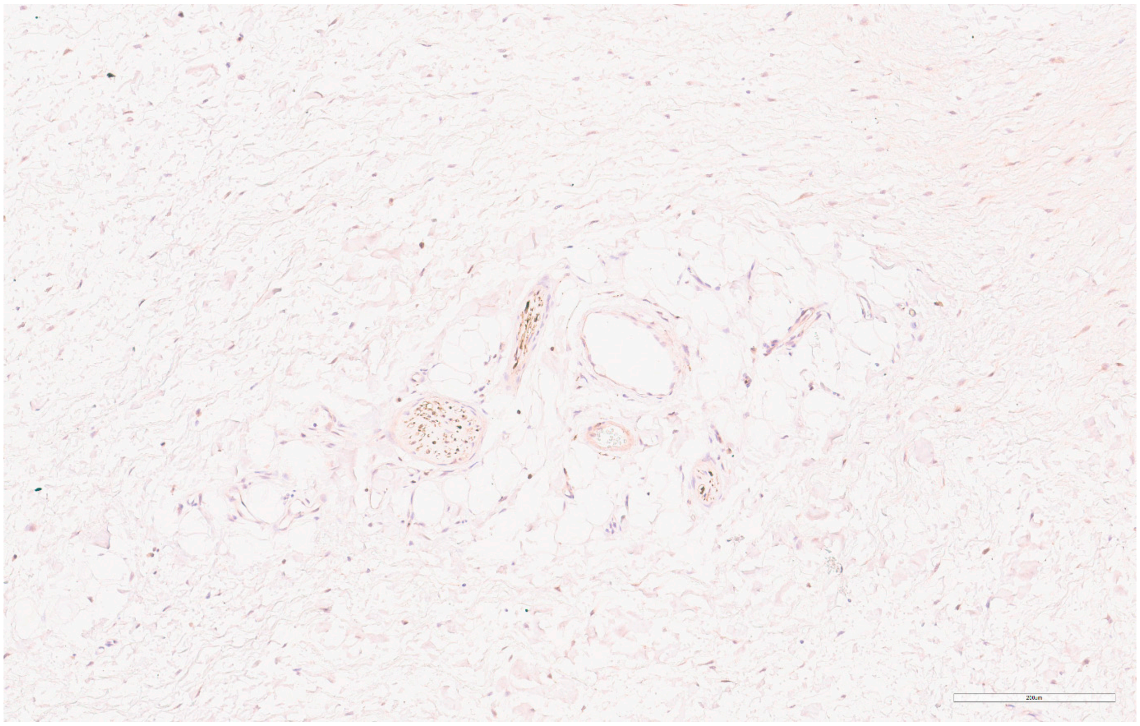

Figure S4. Representative NSE immunohistochemical appearance of morphologically preserved vocal fold tissue. The image illustrates NSE-positive neural elements within the deep lamina propria (DLP) of the human true vocal fold. Brown cytoplasmic staining highlights neural profiles distributed within the stromal compartment, supporting the presence of neural components associated with the vocal fold microenvironment. The analyzed region was selected beyond accepted oncological safety margins and was histopathologically re-evaluated to exclude tumor infiltration or reactive alterations. Original magnification: ×200.

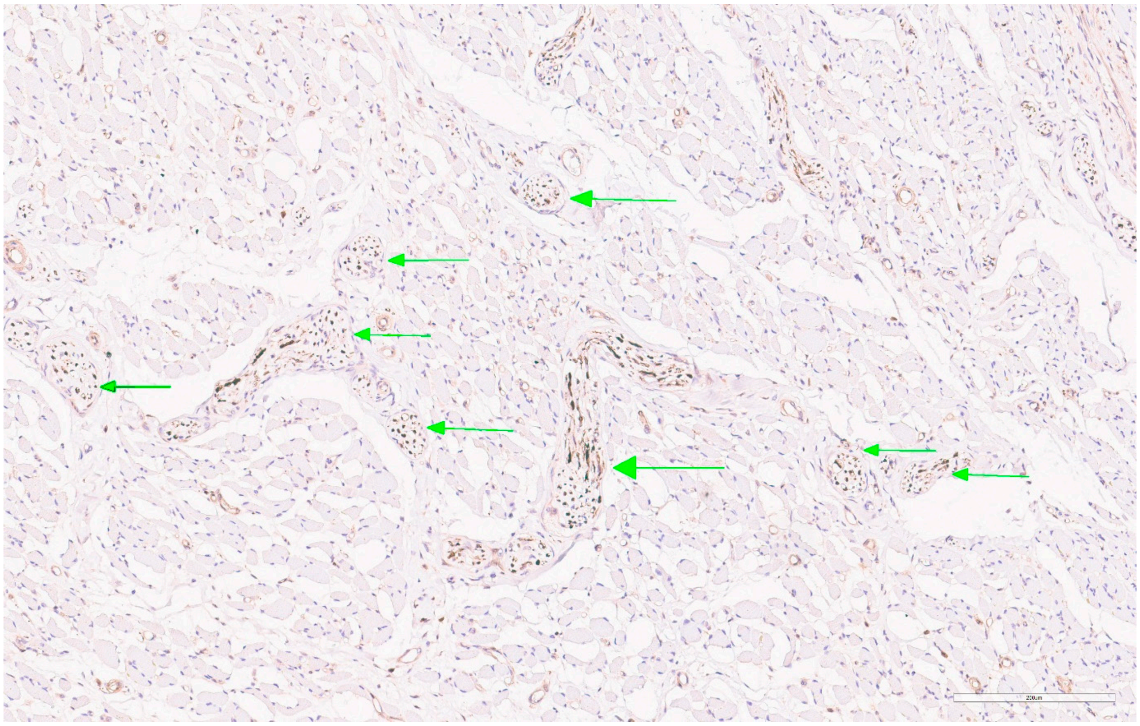

Figure S5. Representative NSE immunohistochemical appearance of morphologically preserved vocal fold tissue. The image demonstrates NSE-positive neural elements within the vocalis muscle (MV) of the human true vocal fold. Brown cytoplasmic staining highlights neural profiles and more organized neural structures distributed among muscular fibers, consistent with the increased neural density observed in the vocalis compartment. The analyzed region was selected beyond accepted oncological safety margins and was histopathologically re-evaluated to exclude tumor infiltration or reactive alterations. Original magnification: ×200.

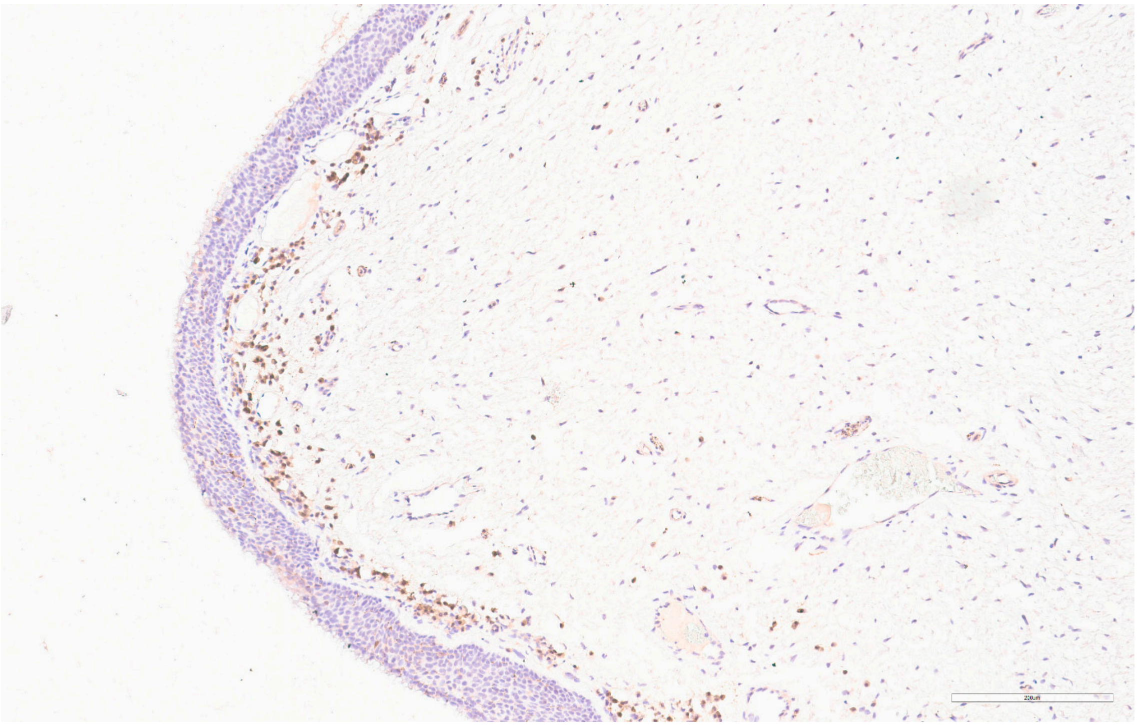

Figure S6. Representative NSE immunohistochemical appearance of morphologically preserved vocal fold tissue. The image illustrates NSE-positive neural elements within the superficial lamina propria (SLP) of the human true vocal fold. Brown cytoplasmic staining highlights delicate neural profiles distributed throughout the superficial stromal compartment, consistent with the regional neural organization observed in the quantitative analysis. The analyzed region was selected beyond accepted oncological safety margins and was histopathologically re-evaluated to exclude tumor infiltration or reactive alterations. Original magnification: ×200.
